# Negative (Workaholic) Emotions and Emotional Exhaustion: Might Job Autonomy Have Played a Strategic Role in Workers with Responsibility during the Covid-19 Crisis Lockdown?

**DOI:** 10.3390/bs10120192

**Published:** 2020-12-15

**Authors:** Paola Spagnoli, Danila Molinaro

**Affiliations:** Department of Psychology, University of Campania Luigi Vanvitelli, 81100 Caserta, Italy; danila.molinaro@unicampania.it

**Keywords:** negative (workaholic) emotions, decision-making autonomy, emotional exhaustion, Covid-19 crisis, managers, freelancers

## Abstract

Decision-making autonomy may have played a crucial role in protecting, or on the contrary, intensifying the onset of negative psychological outcomes for workers with roles with responsibilities during the lockdown due to the Covid-19 crisis. The present study analyzed the emotional dimension of workaholism in relation to emotional exhaustion, considering decision-making autonomy as a moderator of this relationship, in a sample of 101 managers and freelancers in the middle of the lockdown in Italy (early April 2020). Results showed that the relationship between negative (workaholic) emotions and emotional exhaustion was moderated by decision-making autonomy and this moderation differed for managers and freelancers. In particular, the results showed that in the target of managers high levels of negative emotional experiences related to workaholism and low decision-making autonomy are associated with higher levels emotional exhaustion, while high levels of emotional experiences linked to workaholism and high decision-making autonomy produced significantly lower levels of emotional exhaustion. On the contrary, low levels of job autonomy were associated to higher level of emotional exhaustion when negative (workaholic) emotions were low for the freelancers. Limitations are related to the limited sample and the cross-sectional nature of the study. Practical implications lie in considering decision-making autonomy as a double-edged sword, such that when low it could be a risk for managers and when high it could be a risk for freelancers.

## 1. Introduction

During the lockdown period due to the COVID-19 crisis, the sudden change in the way of working to “forced remote working from home” may have caused negative emotional experiences of various kinds among workers. Although it has been demonstrated that virtual work might be related to some positive outcomes, such as improving performance, cutting the costs of “home–work–home” travelling, saving time and organizational resources, and increasing employee satisfaction [1,2], the “sudden forced remote working from home” might have found workers and organizations unprepared, increasing the risks for the onset of negative psycho-social and health consequences such as psychological stress, social isolation, work–family conflict, and difficult time-management [3].

This study focused on workers with responsibility roles such as managers and freelancers. Managers are responsible for their subordinates, as managers have to coordinate their subordinates’ work goals and efficacy in achieving them, while freelancers are responsible on their own work goals and job performance and are not hinged in a specific organization, thus, they are not influenced by the organization’s cultures. We included just managers and freelancers because we wanted to focus on the working categories characterized by higher autonomy. Working remotely and necessarily from home created conditions, such as the interruption of the work routine, the impossibility of completing work goals, and the sudden need to share work spaces and equipment with one’s family, which may have favored a decrease in the job autonomy and the onset of feelings of guilt, anxiety, frustration, and anger. These emotional experiences, exacerbated by individual tendencies to work excessively and compulsively, and by the limited autonomy in the decision-making process, might have led to increase the possibility of experiencing emotional exhaustion.

The individual tendency of working excessively and compulsively is termed “workaholism” [4]. Workaholic employees are “persons whose need for work has become so excessive that it creates noticeable disturbance or interference with his bodily health, personal happiness, and interpersonal relations, and with his smooth social functioning” [5] (p. 4).

Workaholics invest a lot of time and energy in their work, without respecting any boundaries between working and private lives. They also work in the evening and weekend, at the cost of other private and family activities and relations. Thus, we believe that workaholics might have been more exposed to negative outcomes due to the deprivation of the normal work conditions and that this experience might have been worse for workers with responsibility roles. Drawing from the Job Demand-Control Theory [6] and from the Demand-Induced Stress Compensation (DISC) model [7], we examined the role of decision-making autonomy in the relationship between negative (workaholic) emotions and emotional exhaustion in a sample of workers with a responsibility role, such as managers and freelancers during the first stage of the Covid-19 crisis, that is the national lockdown. 

### 1.1. Negative (Workaholic) Emotions and Emotional Exhaustion

Recent studies reported a series of negative workaholism’ health consequences, such as job stress and burnout [8,9,10], psychophysical strain [11], low sleep quality and daytime sleepiness [12], anxiety/insomnia, somatic symptoms and social dysfunction [13], and work–family conflict [10,14,15]. In the literature, negative consequences in the working context are also reported, such as counterproductive work behavior [16] and reduced job satisfaction [9].

The key attributes of workaholism based on the review of the several conceptualizations and measures of workaholism included an uncontrollable inner compulsion to work [5,17,18], persistent, uncontrollable thoughts about work [4,19], negative emotions when not working [19,20,21], and excessive work behaviors [4,22].

Drawing from the previous models proposed in the literature, and adopting a comprehensive and critical view, one of the most recent conceptualizations depicts workaholism as a multidimensional phenomenon capturing all of its nuances [23]. Accordingly, Clark and colleagues [23] formally defined workaholism as a multidimensional construct comprised of: (1) an inner pressure or compulsion to work (i.e., motivational dimension), (2) persistent, uncontrollable thoughts about work (i.e., cognitive dimension), (3) feeling negative emotions when not working or when prevented from working (i.e., emotional dimension), and (4) excessive working that goes beyond what is required and expected (i.e., behavioral dimension). Clark and colleagues [23] claimed that across different situations and over time, certain dimensions of workaholism may be more or less predictive of various individual, organizational, and relationship outcomes. Accordingly, we believe that the emotional dimension of workaholism could have played a very meaningful role during the Covid-19 lockdown given that workers were suddenly deprived from the normal conditions of working and, thus, might have experienced a negative emotional accumulation leading to emotional exhaustion. Emotional exhaustion is the core dimension of burnout, that is a psychological syndrome involving emotional exhaustion, depersonalization, and a diminished sense of personal accomplishment that occurred among various professionals who work with other people in challenging situations [24]. The relationship between workaholism and burnout has been extensively studied in the literature, and many studies have found that workaholism is positively related to burnout [25]. This makes sense given that workaholics often work longer than others, with little opportunity to engage in leisure activities which may serve as crucial means of rest and recovery [19,26]. Although the relationship between workaholism and emotional exhaustion is established in the literature, and the previous contribution on the relationship between the emotional dimension of workaholism and emotional exhaustion provided a meaningful result [23], specific contributions on the particular epidemiologic crisis context that we are experiencing are still lacking. We believe that workers with workaholic tendencies might have experienced more negative emotions than others workers due to the serious restrictions that were imposed by the Italian government during the lockdown. In particular, during lockdown in Italy, only essential activities were allowed, and only indispensable shops were permitted to be open. Individuals had permission to leave their homes just for demonstrated necessities, such as for health reasons, shopping for basic needs, and for working (if working from home was not possible). Most of the firms had to close down because just fundamental goods and services were allowed to be produced and delivered. The sudden forced working from home during the lockdown has caught many workers and firms unprepared. Remote working was the only way of continuing working due to the confinements for many workers who, on one hand, were happy to be protected at home from the contagious virus, but, on the other hand, had to cope with several changes in their working habits and in their life in general. For example, some of these changes regarded the overlap between work and private life and the consequent possible role conflict and family related interruptions while working or vice-versa, and the massive use of information and communication technology with the risk of technology invasion and technostress [27,28]. Workaholic workers might have experienced more negative reactions to the work-life conflict because they are addicted to work and any hinderances to work, such as family duties or sharing the techno-devices for working with the other components of the family, might have been harder for them. Moreover, the massive use of the new technologies and the risk for technostress might have forced the workers to learn quickly how to work efficiently in this new work context at the expense of the work task performance. Workaholics might have found this delayed performance pace as an unacceptable standard, experiencing more negative emotional reactions. The accumulation of all of these negative emotions, such as feeling of guilt, anger, anxiety, and frustration might have led the workaholics to emotional exhaustion. Thus, we put forward the first hypothesis:

**Hypothesis** **1 (H1).** *Negative (workaholic) emotions are positively related to emotional exhaustion.*

### 1.2. The Moderated Role of Job Autonomy in the Relationship between Negative (Workaholic) Emotions and Emotional Exhaustion

Job autonomy reflects the extent to which a job allows freedom, independence, and discretion to schedule work, make decisions, and choose the methods used to perform tasks [29,30]. Job autonomy has been heavily researched in the areas of job design and decision-making, and a seminal meta-analysis in the eighties revealed that a high level of perceived autonomy was associated with high levels of job satisfaction, commitment, involvement, performance, and motivation, whereas a low level of job autonomy was associated with physical symptoms, emotional distress, role stress, absenteeism, and intent for turnover [31]. Recent studies also supported, on one hand, the positive association between a high level of job autonomy and psychological well-being [32], work engagement [33], and, on the other hand, the negative association between low levels of job autonomy and depressive mood [34], anxiety [35], and burnout [36]. However, there are also some inconsistencies in the available evidence, reporting that job autonomy could be considered a double-edged sword [37], and that too much job autonomy may be detrimental to employees [38]. 

Job autonomy represents a form of control on the job, that is the degree of decision authority, and it constitutes one of the core dimensions of the Job Demand-Control (JDC) theory [6]. Decision authority assesses the organizationally mediated possibilities for workers to make decisions about their work (autonomy) [39]. According to the strain hypothesis of the JDC model, employees working in a high-strain job (high demands-low control) experience the lowest well-being, whereas the buffer hypothesis states that control can moderate the negative effects of high demands on well-being. Thus, in the same vein of the JCD theory, we considered job autonomy in the decision-making a key variable in the context of the forced remote working due to the lockdown for the workers with responsibilities, such as managers and freelancers. Following the JCD theory, a further conceptualization on the onset of job stress was proposed in the literature called Demand-Induced Stress Compensation (DISC) model [7]. The DISC model attempted to overcome some of the weaknesses of the JCD theory, such as, the lack of particular (psychological) mechanisms of how (specific) job demands should be compensated by (specific) job resources and the neglected focus on the emotional components of the stress development process, among others. Therefore, drawing from the DISC model, in the current study we aimed at testing a particular compensation stress model starting from a component of specific emotional demands, that is the negative (workaholics) emotions experienced during the lockdown, and its relation to its corresponding emotional outcome, namely, emotional exhaustion. In this relationship, we aimed at testing the moderating role of job autonomy in the decision-making process. 

Unfortunately, during the forced lockdown due to the Covid-19 crisis, the degree of the workers’ autonomy was compromised due to the serious restrictions that were imposed. Even the freelancers and the managers, who are among the professional categories that are more characterized by job autonomy, might have perceived their job autonomy to be drastically reduced. For example, managers had to face the changed communications modalities with their subordinates and, in this context, might have found difficulties in the re-design of their job goals. In fact, the scenery in the society was so unpredictable that every strategy had a high risk of failing. Thus, in general the decision-making process stalled and the managers might have felt that they could not take any decision in autonomy, given that all the decisions were strictly hierarchical and to be taken by the organization chain of command following the restriction policies issued by the Italian Government. On the other hand, freelancers also could not count on their usual degree of autonomy because any decision about their job had to be taken considering the possible lack of goods and services due to the firms shut-down. Thus, it is reasonable to assume that those workers with workaholic tendencies who experienced negative (workaholic) emotions were likely to experience emotional exhaustion, since they had less autonomy in deciding how to manage their work.

However, it is likely that those managers and freelancers, who had maintained a certain degree of autonomy in the decision-making process, would have perceived less hinderances in achieving their job goals and, thus, less emotionally exhausted. 

Thus, according to the view that job autonomy may constitute a “resource” that could compensate the negative effect of negative (workaholic) emotions on the onset of emotional exhaustion, we put forward the following hypothesis:

**Hypothesis** **2 (H2).** *Job autonomy in decision-making moderated the relationship between negative (workaholic) emotions and emotional exhaustion, such that higher levels of negative (workaholic) emotions and low job autonomy are related to higher levels of emotional exhaustion, whereas higher levels of negative (workaholic) emotions and high job autonomy are related to lower levels of emotional exhaustion*.

**Hypothesis** **3 (H3).** *The same moderating effect of job autonomy in decision-making in the relationship between negative (workaholic) emotions and emotional exhaustion was expected both for the group of managers and the group of freelancers.*

## 2. Materials and Methods

### 2.1. Participants 

The participants were 101 individuals (45.5% women) working as managers (43.6%) and freelancers (56.4%). Most of them worked in the private sector (77.7%). Their age ranged from 21 to 64 years old (Mean = 41.02; Standard Deviation = 10.32) and their educational level was high school (33.7%) and bachelor or master degree (65.3%). 

### 2.2. Procedure

Participants were asked to respond to an online questionnaire during the lockdown due to the Covid-19 crisis in April in Italy. The data collection was carried out by graduating students of work and organizational psychology courses as part of their master degree thesis. Students were first trained on how to present the study and its objectives to potential participants, including how to take advantage of the snowball sampling technique. Subsequently, students were asked to identify acquaintances in their social network and to propose them to take part in a study on work-related health and well-being.

### 2.3. Measures

Negative (Workaholic) Emotions: These were measured by four items of the emotional sub-dimension of the Multidimensional Workaholic Scale (MWS) [23]. The “negative (workaholic) emotions” represent a sub-dimension of the MWS. Clark and colleagues argued that workaholism is a multi-faceted phenomenon and that its subdimensions might be considered in different situations because they are related to its different levels. In particular, in the current study we focused on the “emotion” level of the workaholism phenomenon and that is why we used only this subdimension. An item example is “I am almost always frustrated when I am not able to work”. Respondents were asked to answer with a scale ranging from 1 = never to 5 = always. Cronbach’s alpha was 0.85.

Decision-Making Autonomy: This was measured by three items of the decision-making autonomy subscale of the Work Design Questionnaire [40]. An example item is: “The job provides me with significant autonomy in making decisions”. Respondents were asked to answer with a scale ranging from 1 = never to 5 = always. Cronbach’s alpha was 0.92.

Emotional Exhaustion: This was measured by the Italian version of the eight items of the emotional exhaustion sub-scale of the Burnout Assessment Tool [41] adapted by Consiglio, Mazzetti, and Schaufeli (unpublished manuscript). An example item is: “Everything I do at work requires a great deal of effort”. Respondents were asked to answer with a scale ranging from 1 = never to 5 = always. Cronbach’s alpha was 0.90.

### 2.4. Ethics

The procedure was in accordance with the standards of the national law of data treatment, which is strictly followed by the University of Campania “Luigi Vanvitelli”. Since there was no medical treatment or other procedures that could cause psychological or social discomfort to participants, who were all healthy adult subjects anonymously involved, additional ethical approval was not required. The research was conducted in line with the Helsinki Declaration [42], as well as the data protection regulation of Italy (Legislative Decree No. 196/2003). Participation in the study was voluntary and not rewarded, while data collection and analysis were anonymous. A cover letter attached to the questionnaire provided information about the study aims, guarantees about anonymity, voluntary participation and data treatment, and instructions for filling out the questionnaire. When agreeing to fill out the questionnaire, all study participants provided their informed consent.

### 2.5. Data Analysis

Zero-order correlations were used to examine associations between variables. A reliability analysis was used to assess the scales’ internal consistencies. The hypotheses concerning direct and moderated effects were tested through conditional process analysis based on Ordinary Least Square (OLS) regression using a bootstrapping technique [43]—a nonparametric resampling procedure that does not assume normality and involves the extraction of several thousand subsamples (5000, in our case) from a dataset. Through bootstrapping, the distribution of effects is empirically approximated and used for calculating confidence intervals. Specifically, the model examined in the current study corresponds to the conceptual model number 3 of Hayes’ templates. 

## 3. Results

Table 1 shows descriptive analysis and zero-order intercorrelations of the variables in this study. Results pointed out that negative (workaholic) emotions were positively and significantly correlated to emotional exhaustion and decision-making autonomy was negatively and significantly related to emotional exhaustion. Table 2 concerns the results of the conditional process analysis on emotional exhaustion. Negative (workaholic) emotions, decision-making autonomy, and their interaction were related to emotional exhaustion, as well as the interaction among negative (workaholic) emotion, decision-making autonomy, and type of job. The type of job was not significantly related to emotional exhaustion, as well as the interactions between negative (workaholic) emotion and type of job and between decision-making autonomy and type of job.

**Figure 1:**
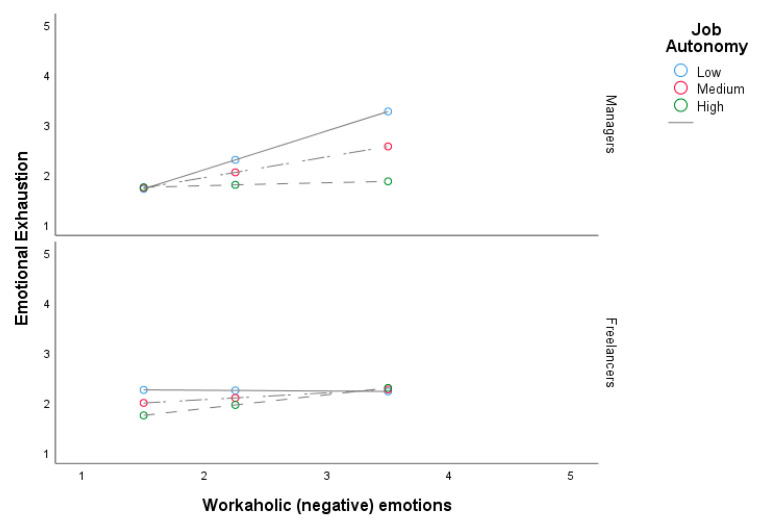
The moderated moderation effect of decision-making autonomy in the relationship between negative (workaholic) emotions and emotional exhaustion for the two groups of employees (managers/freelancers).

Following Hayes [43], the values of negative (workaholic) emotions were observed at the 16th, 50th, and 84th percentile of decision-making autonomy. In the managers’ plot displayed in Figure 1 when negative (workaholic) emotions are high and decision-making autonomy is low, emotional exhaustion is significantly higher than when decision-making autonomy is high. As far as the simple slopes are concerned results pointed out that just the simple slopes concerning low and medium autonomy for the managers and high autonomy for the freelancers were statistically significant, with the highest effect for the combination of low levels of decision-making autonomy in the group of managers (B = 0.77, LLCI = 0.42, ULCI = 1.13). However, a test of the conditional interaction of negative (workaholic) emotion and decision-making autonomy at the two levels of the job type revealed that the effect (B = 0.28, *p* < 0.05) was significant only for the managers, whereas was not significant for the freelancers (B = −0.26, *p* = 0.10). Thus, we can conclude that low decision-making autonomy had an enhancing effect between negative (workaholic) emotion and emotional exhaustion in the group of managers, and thus our hypotheses were just partially supported.

## 4. Discussion

The current study, drawing from the DISC model [7], provided supporting evidence of the enhancing effect of low levels of decision-making autonomy in the relationship between negative (workaholic) emotions leading and emotional exhaustion in the managers group. Moreover, contrary to the expectations, a further negative enhancing effect was found for high level of job autonomy in the relationship between low levels of negative (workaholic) emotions and emotional exhaustion. Thus, results seem to support the double-edged sword effect of job autonomy, which would not be positive in absolute, but its effect could vary according to the job characteristics [38]. Although we expected decision-making autonomy to play the same role both for the managers and for the freelancers, we found a different role played in the two groups. 

We included managers and freelancers in the study because they represent job categories usually characterized by a high level of job autonomy and, as both managers and freelancers were forced to work remotely during the lockdown, we wanted to explore if the protective role of job autonomy was effective for both of them. A reason for the negative enhancing role of high level of job autonomy for the freelancers might be related to the fact that, workaholics or not, their job role might imply a high degree of risk for strain in general because freelancers earn if they can work, that is, they cannot count on financial income in the event of a crisis, because they are not protected from a contractual point of view. Particularly, during the Covid-19 crisis, the lockdown imposed by the Government guaranteed just a minimum financial subsidy to the freelancers, and with the uncertainty of the future due to the epidemiological emergency the perception of job insecurity increased, and it must have been really hard to keep calm and focus at work. However, it has to be said that the freelancers’ category includes different types of freelancers (lawyers, architects, web designers, journalists, psychologists, etc.). For example, there are freelancers who worked on temporary contracts with organizations, or there are freelancers who do private consultancy. It is likely that the job role of the freelancers who work with temporary job contract in organizations is characterized by less job autonomy than the freelancers who do private consultancy. Moreover, as suggested by de Jonge and Schaufeli [44] in the case of low need for autonomy, high job autonomy could be positively related to emotional exhaustion, whereas for high need for autonomy the resulting relationship between job autonomy and exhaustion could be curvilinear (representing an inverted U-shape). Thus, it is possible that in the context of the Covid-19 epidemiologic crisis, the freelancers need for autonomy was decreased and it is likely that their priorities shifted from more autonomy = more uncertainty towards the wish of less autonomy = less uncertainty.

On the other hand, during the lockdown managers were concerned with organizing other workers work by adopting new ways of communication through the new media, and, thus, the decision-making process was very important in order to let the work process proceed. This was really challenging for the managers, who had to consider the command chain when taking a decision in a crisis period. Thus, it is possible that managers, especially those with workaholic tendencies, perceived to have their hands tied with their need for autonomy increased and, as a consequence, exacerbating their negative emotional states.

Although this study provided interesting evidence, some limitations should be taken into account. First, the limited and convenience sample along with the cross-sectional nature of the study. Thus, a wider and representative sample is recommended in future studies and also a longitudinal method should be used to strengthen the results. Moreover, future studies should also consider other job categories to verify the protective role of high levels of job autonomy. Furthermore, personality variables might be considered as covariates in future studies to control the effect that personal disposition could have in this mechanism. As we previously mentioned, the need for autonomy, should be investigated as a further moderating variable. Additionally, freelancers are a job category that includes several different type of job conditions, and maybe differences between the condition of a temporary worker for an organization and a private consultant should be taken into consideration. Finally, future studies would also benefit from a mix-method approach. Specifically, qualitative studies should be conducted to deepen the results provided by quantitative studies. For example, focus groups or individual interviews might be conducted to better understand the perception of job autonomy and its related emotional dimensions in the two targets of workers.

Despite these limitations, the current study also provided some clues for useful practical implications to be considered during periods of epidemiologic crisis. Since the role of the managers represents a key role for the work processes, they should preserve a certain degree of autonomy in the decision-making process. Thus, a dedicated coaching service might be provided by the organization to the managers in order to promote their wellbeing and reduce the risk for detrimental health outcomes, such as emotional exhaustion, which is a core dimension of burnout syndrome. Moreover, since the epidemiologic risk still exists and might also exists in the future, organizations should foresee specific plans to help managers to cope in times of crises. Freelancers, on the other hand, could be made aware of the risk of having “too much” decision-making autonomy, as this autonomy could lead them to sacrifice the time and space dedicated to the rest of their lives. For example, professional associations could raise awareness on the subject through training sessions for a more correct and sustainable management of their work. Finally, policies during crisis times should consider more financial support for freelancers since their feeling of job insecurity, which normally is compensated by the high degree of job autonomy, was amplified with all of the negative consequences that this may bear in the whole economic Italian system. 

## Figures and Tables

**Table 1:** Descriptives and intercorrelations of the study variables.

	Mean	St. Dev.	Gender	Age	Negative (Workaholic) Emotions	Decision-Making Autonomy
Age	41.03	10.33	0.17			
Negative (workaholic) Emotions	2.48	0.93	−0.14	0.03		
Decision-making autonomy	3.98	0.91	−0.23 *	0.18	0.10	
Emotional exhaustion	2.12	0.74	0.09	−0.27	0.29 **	−0.24 **

Note: ** = *p* < 0.001; * = *p* < 0.05 St.Dev. = Standard Deviation.

**Table 2:** Conditional process analysis on emotional exhaustion.

Variables	B	LLCI	ULCI	R^2^
Outcome: Emotional Exhaustion				0.30
Negative (workaholic) emotions	0.57	0.17	0.96	
Decision-making autonomy	0.45	−0.77	−0.13	
Negative (workaholic) emotions * decision-making autonomy	−0.61	−1.03	−0.19	
Type of job	−0.01	−0.14	0.12	
Negative (workaholic) emotion * type of job	−0.15	−0.30	0.01	
Decision-making autonomy * type of job	0.11	−0.04	0.26	
Negative(workaholic) emotions * decision-making autonomy * type of job	0.25	0.07	0.43	
Gender	−0.07	−0.35	0.21	
Age	−0.02	−0.03	−0.01	
Moderated effect of workaholism on emotional exhaustion				
Low autonomy/managers	0.77	0.42	1.13	
Low autonomy/freelancers	−0.01	−0.35	0.32	
Medium autonomy/managers	0.42	0.16	0.67	
Medium autonomy/freelancers	0.13	−0.04	0.31	
High autonomy/managers	0.06	−0.33	0.45	
High autonomy/freelancers	0.28	0.01	0.56	
